# Supplementary material for: Advanced Resistance Studies Identify Two Discrete Mechanisms in Staphylococcus aureus to Overcome Antibacterial Compounds that Target Biotin Protein Ligase
Source: Antibiotics (Basel). 2020 Apr 6;9(4):165. doi: 10.3390/antibiotics9040165 (PMC7235819; doi:10.3390/antibiotics9040165)
Supplement: Supplementary file 1 [file antibiotics-09-00165-s001.pdf]

Supporting Information

Advanced Resistance Studies Identify Two Discrete Mechanisms in *Staphylococcus aureus* to Overcome Antibacterial Compounds that Target Biotin Protein Ligase

Andrew J. Hayes[1, 5]†, Jiulia Satiaputra[1, 6]†, Louise M. Sternicki[1,7], Ashleigh S. Paparella[1, 8], Zikai Feng[1, 9], Kwang J. Lee[2], Beatriz Blanco- Rodriguez[2], William Tieu[2, 10], Bart A. Eijkelkamp[1,11], Keith E. Shearwin[1], Tara L. Pukala[2], Andrew D. Abell[2,3,4], Grant W. Booker[1] and Steven W. Polyak[1, 12] *

[1] School of Biological Sciences, University of Adelaide, South Australia 5005, Australia

[2] School of Physical Sciences, University of Adelaide, South Australia 5005, Australia

[3] Centre for Nanoscale BioPhotonics (CNBP), University of Adelaide, Adelaide, South Australia 5005, Australia

[4] Institute of Photonics and Advanced Sensing (IPAS), School of Biological Sciences, University of Adelaide, South Australia 5005, Australia

[5] Present address: School of Biomedical Sciences, The Peter Doherty Institute for Infection and Immunity, The University of Melbourne and The Royal Melbourne Hospital, Melbourne, Victoria 3000, Australia.

[6] Present address: Harry Perkins Institute of Medical Research, The University of Western Australia, Perth, Western Australia, 6009 Australia

[7] Present address: Griffith Institute for Drug Discovery, Griffith University, Nathan, Queensland 4111, Australia

[8] Present address: Albert Einstein College of Medicine, New York City, New York, 10461 United States of America

[9] Present address: School of Pharmacy and Pharmacology, The University of Tasmania, Hobart, Tasmania, 7005 Australia

[10] Present address: South Australian Health and Medical Research Institute, Adelaide, South Australia, 5000 Australia

[11] Present address: College of Science and Engineering, Flinders University, Australia

[12] Present address: Health and Biomedical Innovation, School of Pharmacy and Medical Sciences, University of South Australia, Adelaide, South Australia, 5001 Australia

† These authors contributed equally

* Correspondence: steven.polyak@unisa.edu.au; Tel.: +61883021603

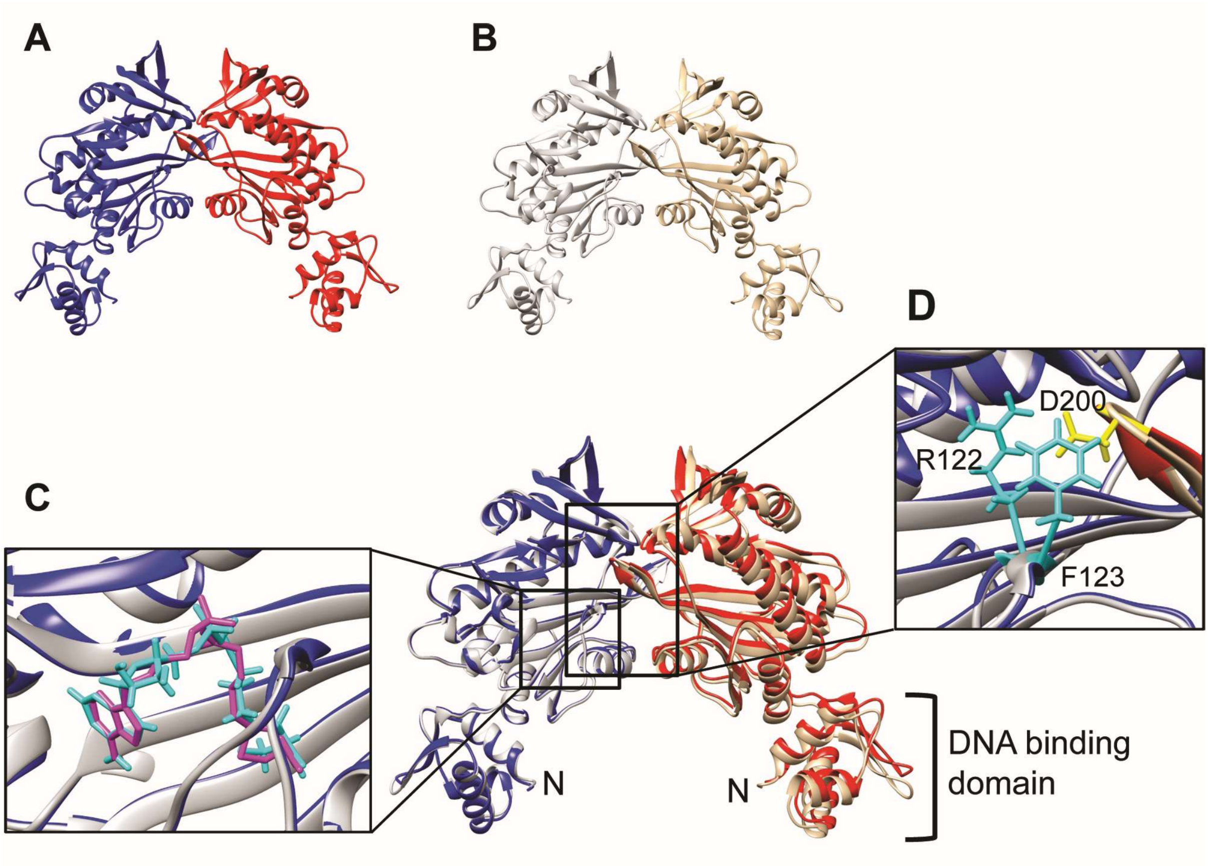

Figure S1: *Sa*BPL structures

Structural overlay of the *Sa*BPL homodimer in complex with A. biotinyl-5′-AMP (subunits in blue and red, PDB ID: 3RIR [1]) and B. BASA (subunits in grey and tan, PDB ID 6ORU [2]). C. Insert panel shows the conserved binding modes of biotinyl-5′-AMP (cyan) and BASA (purple). D. Inter-subunit residues involved in homodimerization – R122 and F123 (cyan) with D200 (yellow) from the opposing subunit.

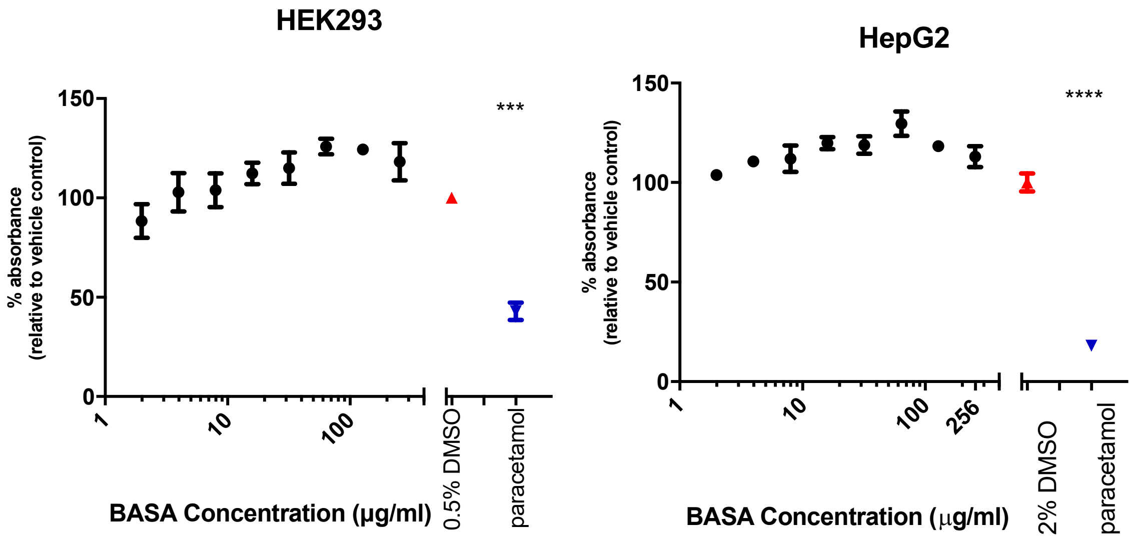

Figure S2: Cytotoxicity of BASA was tested against HEK293 and HepG2 cell lines.

The WST-1 reagent was used to detect metabolic activity after 48 hours of treatment with varying concentrations of BASA. No significant toxicity was observed at any concentration compared to the DMSO vehicle controls (red upward triangles). Paracetamol (blue downward triangles) at 6.25mM and 25mM, respectively, exhibited >60% reduction of growth for the two cell lines (*** = $p<0.001$).

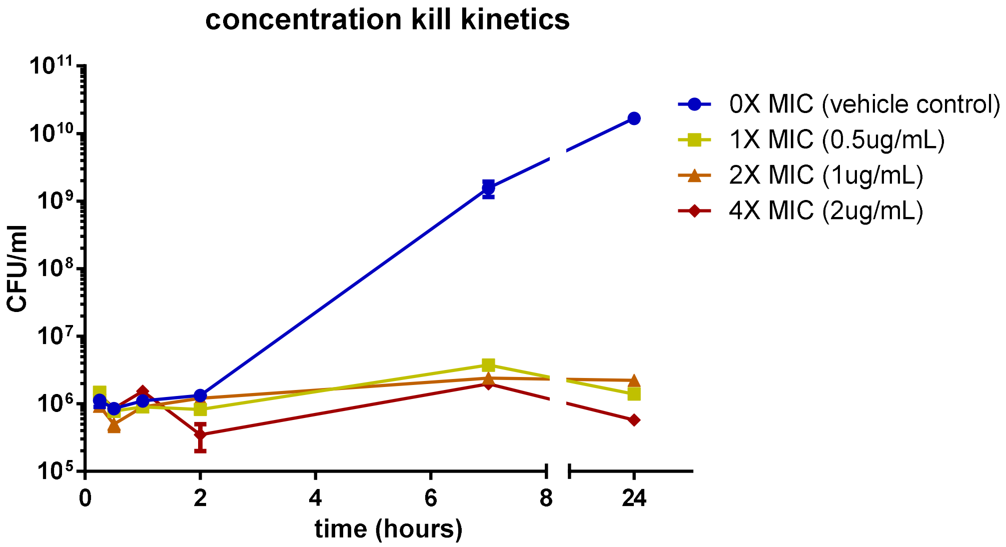

Figure S3: Concentration kill kinetics.

S. aureus ATCC49775 was grown in the presence of no compound (blue), or BASA at 1× MIC (yellow squares), 2× MIC (orange triangles), 4× MIC (red diamonds). Viable cells were enumerated at the end of the treatment to determine if killing was observed.

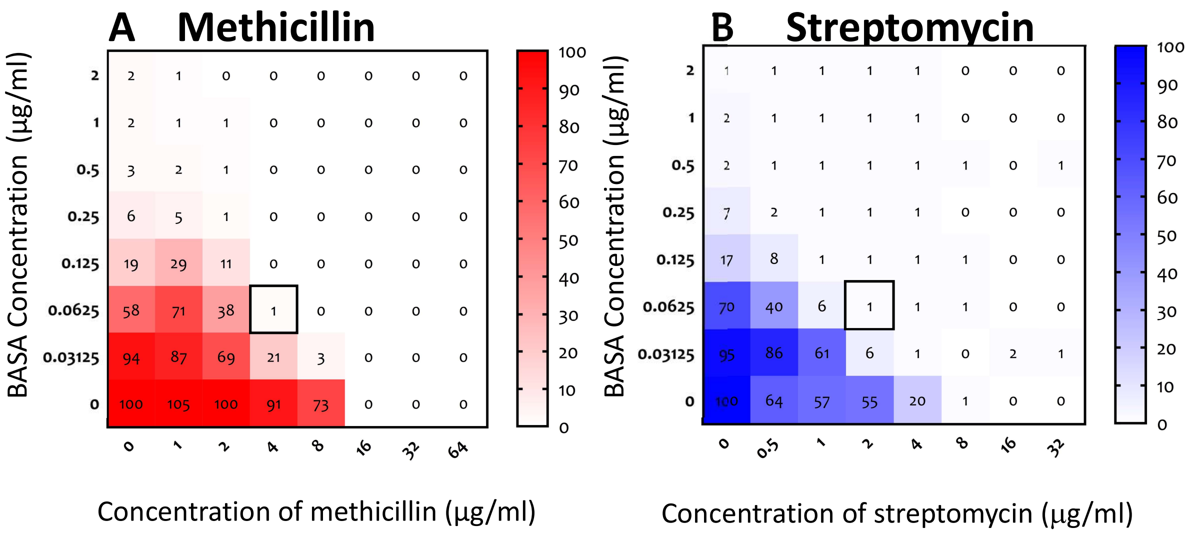

**Antibacterial agent**	**Class**	**Mechanism of action**	**∑ FIC with BASA**	**Synergy**
Synergistic Activity				
Methicillin	β-lactam	Cell wall synthesis	0.375	Yes
Streptomycin	Aminoglycosides	Protein synthesis	0.375	Yes
No Synergistic activity				
Vancomycin	Glycopeptides	Cell wall synthesis	0.625	No
Daptomycin	Lipopeptides	Cell membrane depolarisation; nucleotide and protein synthesis	0.625	No
Erythromycin	Macrolides	Protein synthesis	0.625	No
Chloramphenicol	Chloramphenicols	Protein synthesis	0.5625	No
Tetracycline	Tetracyclines	Protein synthesis	0.5625	No

Figure S4: Synergism of BASA with various antibiotics.

Checkerboard assays were performed upon *S. aureus* ATCC 49775 with varying concentrations of BASA and A. methicillin or B. streptomycin. Heat maps show relative growth as a percentage of untreated cells (100%). Absorbance ≤5% was classified as optically clear. The boxed well shows the lowest concentration of the two compounds that inhibited growth, and these conditions were used to calculate the fractional inhibitory concentration (∑FIC). Experiments were repeated in biological triplicates.

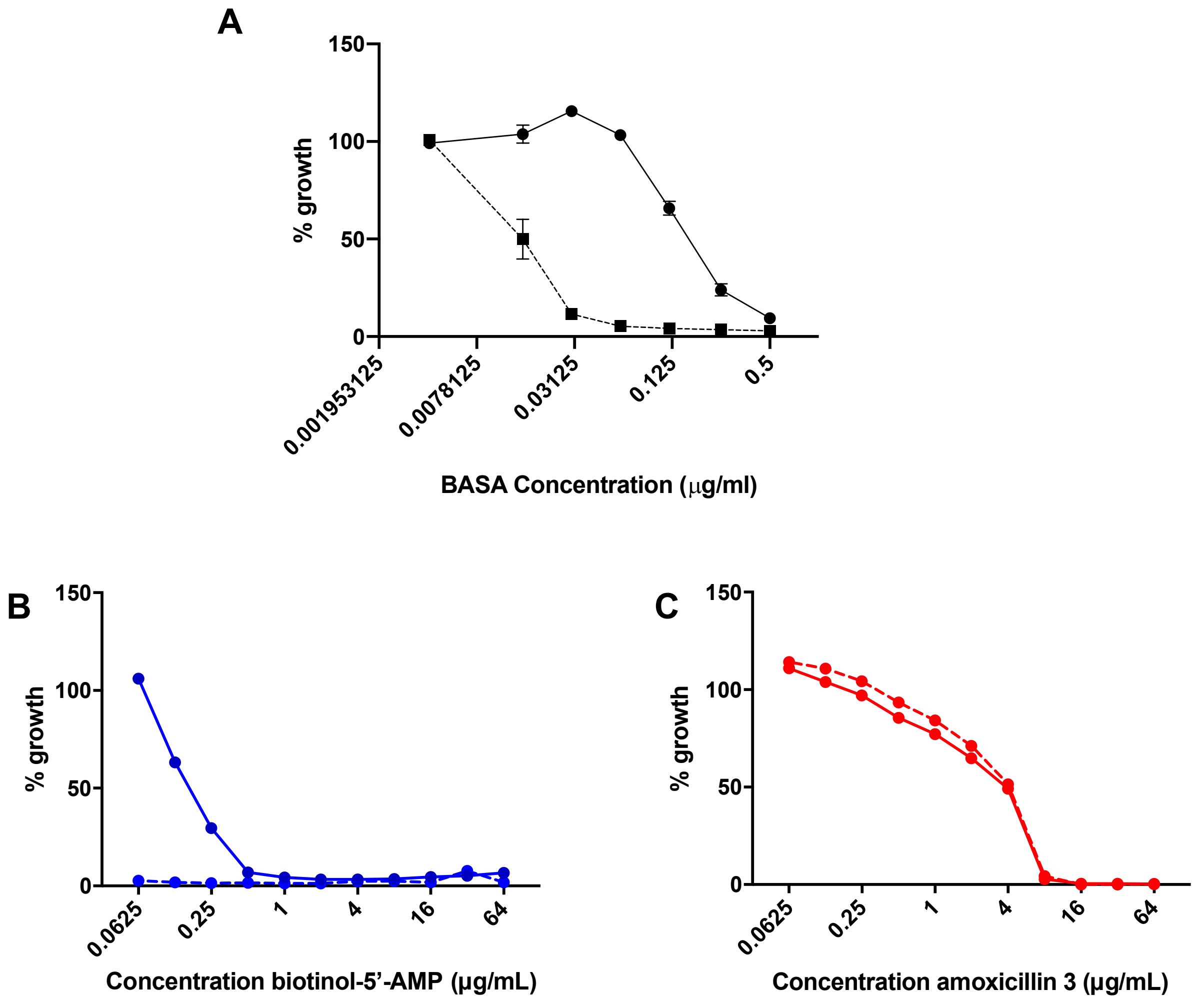

Figure S5: Mechanism of action studies in *S. aureus***.**

A. Susceptibility to BASA for *S. aureus* RN4220 harbouring pCN51-NcoI control (dashed lines) and the pCN51-BPL overexpression plasmid (solid lines). Susceptibility to B. known BPL inhibitor biotinol-5′-AMP and C. non-BPL targeting antibiotic amoxicillin. Assays were performed in triplicate and normalised to no compound control.

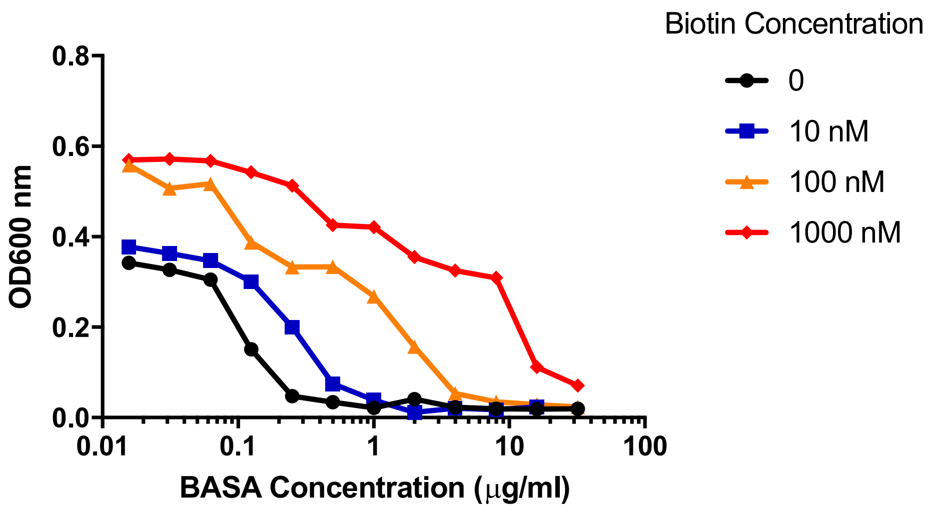

Figure S6: Effect of increased exogenous biotin concentration on the antimicrobial activity of BASA against *S. aureus* ATCC 49775.

Antimicrobial susceptibility assays were performed using varying BASA concentrations with bacteria grown in Mueller Hinton broth supplemented with either no addition biotin (black circles), 10 nM biotin (blue squares), 100 nM biotin (yellow triangles) or 1 μM biotin (red diamonds).

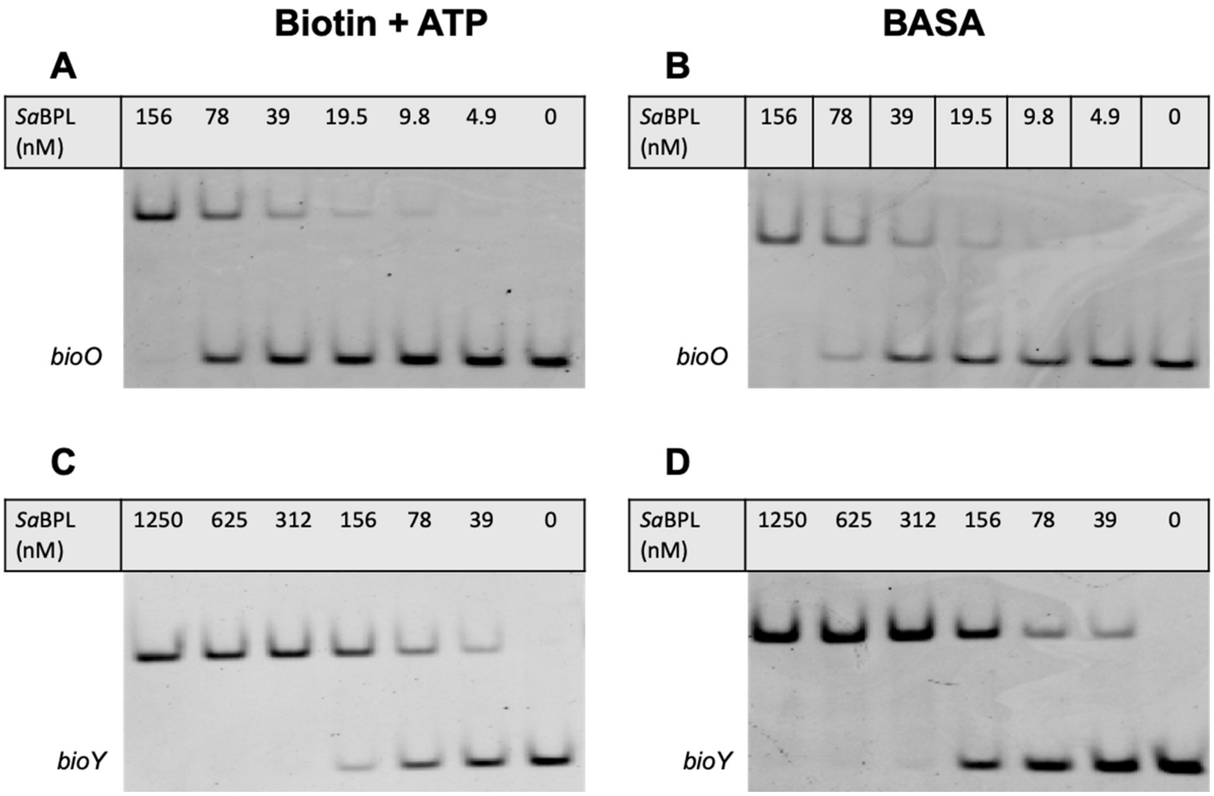

Figure S7: *Sa*BPL in complex with BASA is competent to bind DNA.

EMSA analysis was employed to measure *Sa*BPL binding to the *bioO* oligonucleotide in the presence of A. biotin and ATP and B. BASA, and the *bioY* oligonucleotide in the presence of C. biotin + ATP and D. BASA. Control reactions for EMSA analysis were prepared by adding 0.1 mM biotin, 1 mM ATP and 2.5% DMSO. These were compared to reactions containing 0.1 mM of compound BASA (prepared in DMSO) with 2.5% DMSO.

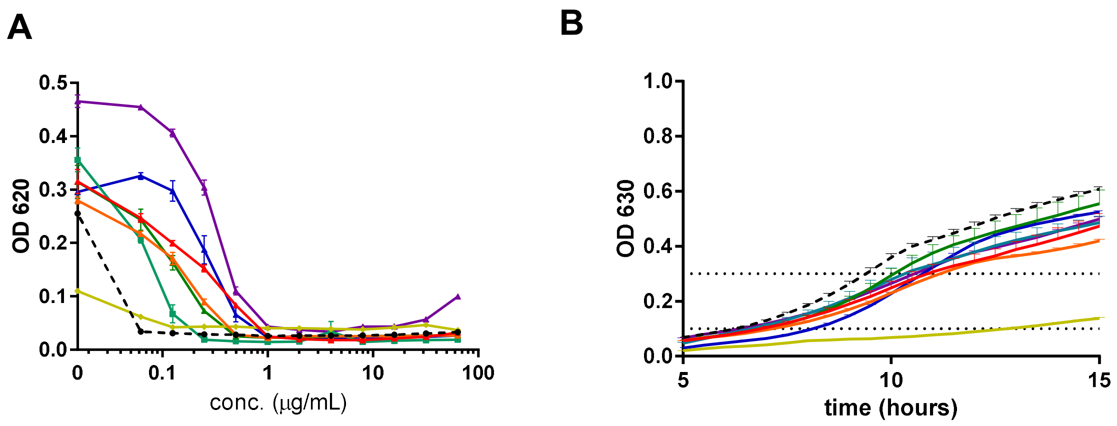

Figure S8: A. Susceptibility of *S. aureus* NCTC8325 derived strains to BASA after serial passage in suboptimal concentrations of the antibiotic for 18 days. B. Growth of strains in Mueller Hinton media measured half hourly. Black dashed line, parental NCTC8325; red, B1; orange, B2; yellow, B3; green, B4; light blue, B5; dark blue, B6; purple**,** B7. Data are the average readings of three independent experiments. Error = SEM.

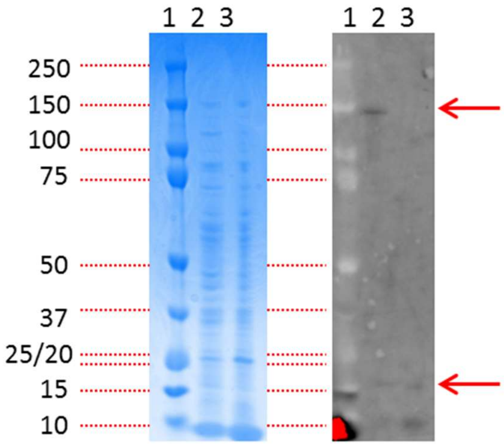

Figure S9: A. Coomassie stained SDS PAGE, and B. Streptavidin blot, of cell lysates from *S. aureus* NCTC 8325 (lane 2), and *S. aureus* B7 (lane 3). In the streptavidin blot both lanes show a band between 15 to 20 kDa (biotinylated ACC subunit ≈ 17 kDa). However the band at approximately 130kDa (PC ≈ 130 kDa) in lane 2 is absent in lane 3, indicating loss of biotinylated pyruvate carboxylase.

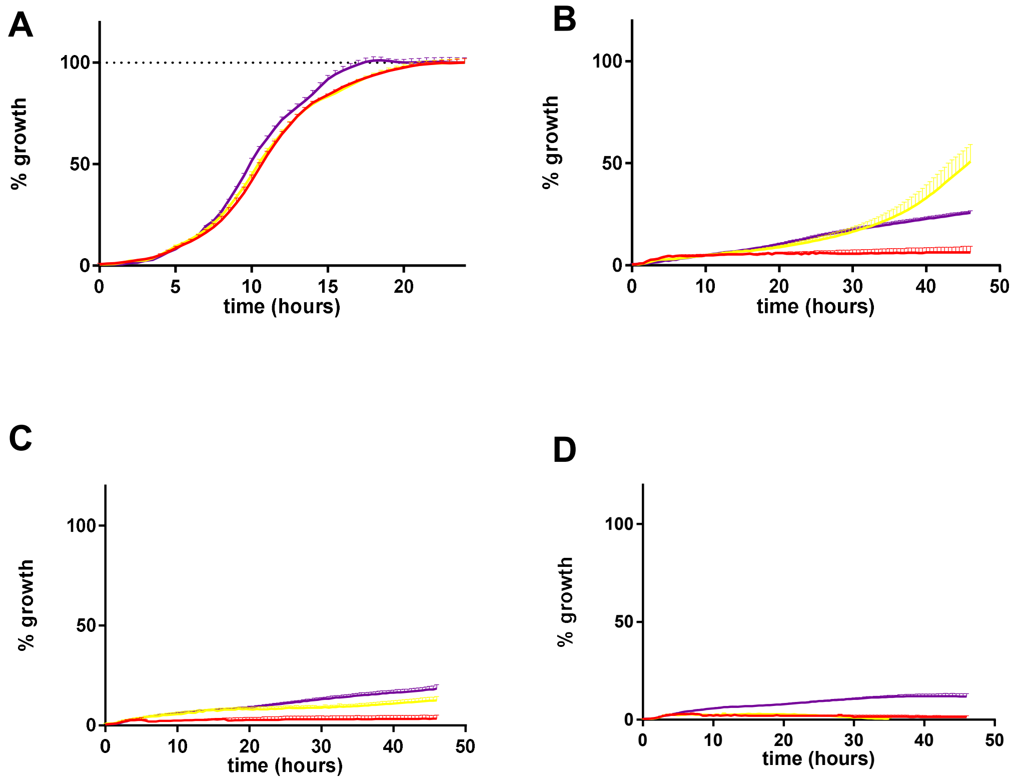

Figure S10: Growth of wild type *S. aureus* USA300 (JE2) (red), and transposon mutagenesis strains Δ*pyc* (yellow) and Δ*yjbh* (purple) deletion mutants over a 48 hour time period in the presence of A. no antibiotic, and BASA at B. 0.5 μg/ml, C. 2 μg/ml, D. 4μg/ml. Growth of strains were normalised to plateau of the untreated control (OD 0.75), which was consistent for all strains. Data are the average of 3 independent experiments. Error = SEM.

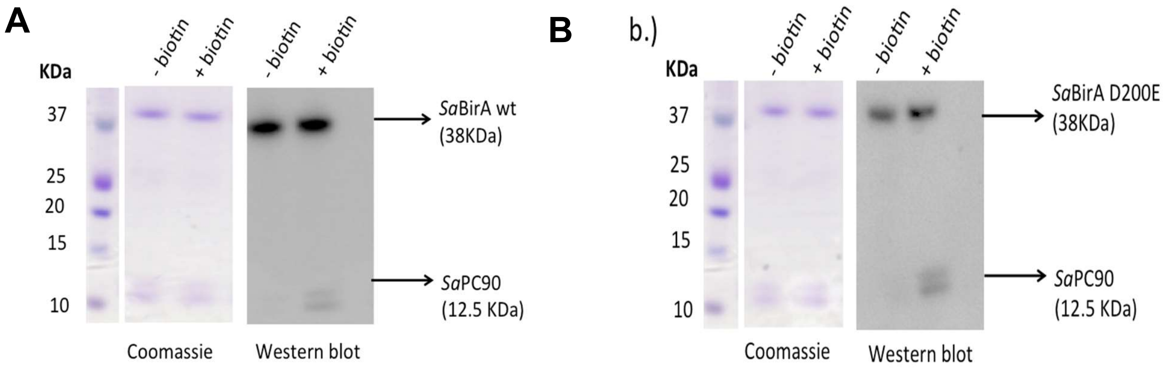

Figure S11. A. wild-type *Sa*BPL and B. *Sa*BPL D200E were both purified in their apo (unliganded) state, with no detectable reaction intermediate bound. Streptavidin-blots were performed to measure biotinylated protein *Sa*PC90, a 90 amino acid fragment of *S. aureus* pyruvate carboxylase, after incubation with *Sa*BPL in the presence and absence of biotin. Coomassie stained gel was used as a loading control.

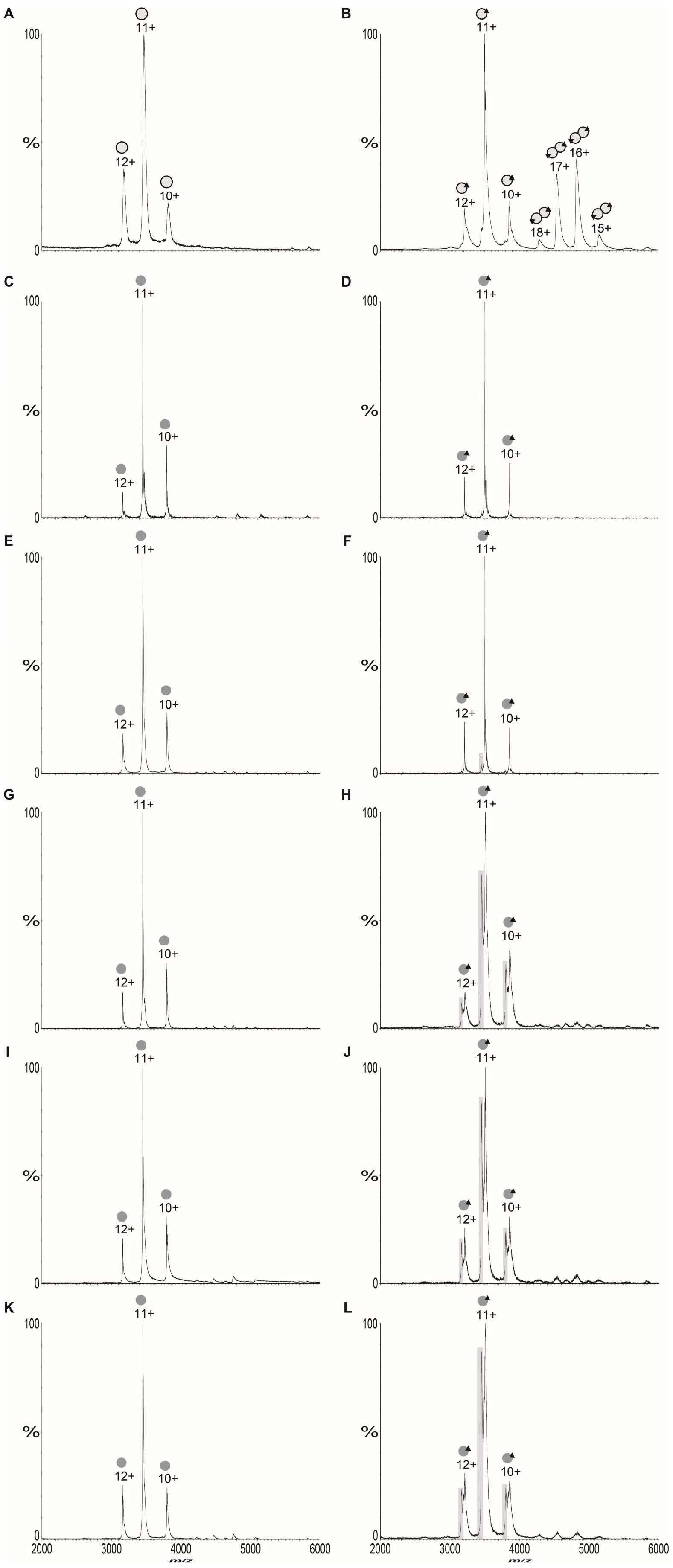
A
B
C
D
E
F
G
H
I
J
K
L
100
%
0
11+
12+
10+
18+
17+
16+
15+
2000
3000
4000
5000
6000
m/z

Figure S12. Native MS reveals *Sa*BPL-D200E is monomeric in the presence and absence of ligands.

nESI-MS to measure the oligomeric state of A. 10 µM apo wildtype *Sa*BPL (outlined light grey circles), B. 10 µM holo wildtype *Sa*BPL, C. 1.4 µM apo *Sa*BPL-D200E (dark grey circles), D. 1.4 µM holo *Sa*BPL-D200E, E. 11.25 µM apo *Sa*BPL-D200E, F. 11.25 µM holo *Sa*BPL-D200E, G. 22.5 µM apo *Sa*BPL-D200E, H. 22.5 µM holo *Sa*BPL-D200E, I. 45 µM apo *Sa*BPL-D200E, J. 45 µM holo *Sa*BPL-D200E, K. 90 µM apo *Sa*BPL-D200E and L. 90 µM holo *Sa*BPL-D200E. Monomer is represented by a single sphere, dimer by conjoined spheres and the presence of biotinyl-5′-AMP by a black triangle.For the preparation of holo enzyme, *Sa*BPL was preincubated with 500 µM biotin, 1 mM ATP and 1 mM $MgCl_2$ (ie saturating concentrations) prior to buffer exchange and MS analysis, as previously described [3].

Table S1: MICs of parental strain NCTC-8325 and the seven BASA-resistant mutant strains for BPL inhibitors and the β-lactam antibiotics methicillin and amoxicillin. Each MIC is the average of three independent experiments.

	**BASA**	**biotinol-5'-AMP**	**BPL223**	**methicillin**	**amoxicillin**
**NCTC-8325**	0.065	1	8	2	16
**B1**	1	>64	64	2	16
**B2**	0.5	>64	>64	2	16
**B3**	0.125	>64	16	4-8	32
**B4**	0.25	>64	>64	2	16
**B5**	0.25	8-16	8	2	16
**B6**	1	>64	>64	2	16
**B7**	4	>64	>64	2	16

Table S2: Strains used in this study and relevant genetic and phenotypic changes associated with BASA resistance. MICs were determined against BASA. Significance for doubling times was determined by ANOVA analysis using Dunnett's multiple comparison test. (*** p<0.001).

Strain	MIC (µg/ml)	Doubling time (minutes)	Genotype
**NCTC 8325**	0.065	51.8 ± 2.1	N/A
**B1**	1	69.2 ± 11.6	*trkA* (+1bp 285/663)(VTAK[95-98]SNC*)<br>*pyc* Δ194bp (2761-2954/3453)<br>SAOUHSC_01981 E20K
**B2**	0.5	64.5 ± 1.42	*pyc* 14bp insertion (1834/3453)<br>Δ94bp intergenic (SAOUHSC_01476 to 01477)<br>presumed silent mutation (no *rsbU*)
**B3**	0.125	193.6 ± 14.3<br>***	*gdpP* H442P<br>*yjbH* Q213-
**B4**	0.25	53.5 ± 5.5	*pyc* G361<br>Δ113bp intergenic (SAOUHSC_01476 to 01477)
**B5**	0.25	68.8 ± 12.1	*greA* Δ111bp<br>SAOUHSC _01504 P15L
**B6**	1	41.9 ± 0.73	*pyc* Δ200bp (917-1116/3453)<br>Δ139bp intergenic (SAOUHSC_01476 to 01477)<br>*rpoβ* P626L<br>SAOUHSC_02984 (*gtfA*-like) R296H
**B7**	4	67.1 ± 0.90	*birA* D200E<br>*pyc* Δ200bp (917-1116/3453)<br>Δ139bp intergenic (SAOUHSC_01476 to 01477)<br>*fmtA* K163
**JE2**	0.5	ND	Parent strain, USA300 JE2
**NR-47297**	4	ND	USA300 JE2 *Δpyc (pyc*::ΦΝΣ)
**NR-47439**	4	ND	USA300 JE2 *ΔyjbH* (*yjbH*::ΦΝΣ) SAOUHSC_00938
**NR-47565**	0.5	ND	USA300 JE2 *ΔfmtA (fmtA*::ΦΝΣ)SAOUHSC_00998
**NR-47335**	0.5	ND	USA300 JE2 *ΔgtfA* (*gtfA*::ΦΝΣ) SAOUHSC_02984
**NR-47331**	0.5	ND	USA300 JE2 *ΔtrkA (trkA*::ΦΝΣ) SAOUHSC_01034

Note. Isolates from B6 and B7 are suspected to be derived from the same intermediate strain, as they contain identical deletions in two separate locations in the genome.

Table S3. Molecular weights of complexes measured by native nano-electrospray ionisation mass spectrometry. Masses are calculated based on the loss of the initiating methionine.

***Sa*BirA Sample**	**Measured MW (Da)**	**Complex components**	**Calculated MW (Da)**
Apo-wild type *Sa*BirA 10 µM	37892	Monomer	37892
Holo-wild type *Sa*BirA 10 µM	38470<br><br>76925	Monomer, biotinyl-5′-AMP bound<br><br>Dimer, biotinyl-5′-AMP bound	38466<br><br>76931
Apo-*Sa*BirA D200E 1.4 µM	37916	Monomer	37905
Holo-*Sa*BirA D200E 1.4 µM	38488	Monomer, biotinyl-5′-AMP bound	38479
Apo-*Sa*BirA D200E 11.25 µM	37919	Monomer	37905
Holo-*Sa*BirA D200E 11.25 µM	38484	Monomer, biotinyl-5′-AMP bound	38479
Apo-*Sa*BirA D200E 22.5 µM	37912	Monomer	37905
Holo-*Sa*BirA D200E 22.5 µM	38488	Monomer, biotinyl-5′-AMP bound	38479
Apo-*Sa*BirA D200E 45 µM	37915	Monomer	37905
Holo-*Sa*BirA D200E 45 µM	38489	Monomer, biotinyl-5′-AMP bound	38479
Apo-*Sa*BirA D200E 90 µM	37915	Monomer	37905
Holo-*Sa*BirA D200E 90 µM	38486	Monomer, biotinyl-5′-AMP bound	38479

Table S4. Oligonucleotides used for cloning in this study.

Primer	Sequence	Function	source
B481	5′ -GGTTGCTAATAATGAAGGTATAGAA GCAATAATATGTGG-3′	D200E mutagenesis	This study
B482	5′ - CCACATATTATTGCTTCTATACCTTCATTATTA GCAACC -3′	D200E mutagenesis	This study
B200	5′-GGTATAGAAGCAATAATATGT GG-3′	Internal BPL sequencing	This study
Lambda P1	5'-GGCATCACGGC AATATAC-3'	attP-λ PCR screening primer	[4]
Lambda P2	5'-ACTTAACGGCTGACATGG-3'	attP-λ PCR screening primer	[4]
Lambda P3	5'-GGGAATTAATTCTTGAAGACG-3'	attP-λ PCR screening primer	[4]
Lambda P4	5'-TCTGGTCTGGTAG CAATG-3'	attP-λ PCR screening primer	[5]

Table S5. Oligonucleotide sequences used for qRT-PCR to measure expression of *bioD* and *bioY* by *Sa*BPL.

Target gene	Primer name	Primer sequence 5'-3'	Source
S. aureus bioD	qSA2716_F	GCAAGGTGTGGTGATACAGG	[6]
	qSA2716_R	ACACGTGGTCATCGAGTTTG	[6]
S. aureus bioY	qSA2552_F	AATGGCAAGCCAGCAACTAC	[6]
	qSA2552_R	GGATTGGTACCGGTAATTCCA	[6]
S. aureus 16s rRNA	qSA0002_F	GAACCGCATGGTTCAAAAGT	[6]
	qSA0002_R	CGTAGGAGTCTGGACCGTGT	[6]

Table S6. Oligonucleotide sequences employed for EMSA analysis of *Sa*BPL DNA binding activity.

Oligo name	Sequence 5'-3'	Description	Source
DS-SaBioO oligo 1	CCTTAAATGTAAACTTTTATAATT AATAAGTTTACATTTAAG	Top strand oligo containing *SabioO* wildtype sequence	[6]
DS-SaBioO oligo 2	CCTTAAATGTAAACTTATTAATTA TAAAAGTTTACATTTAAGG	Bottom strand oligo containing *SabioO* wildtype sequence	[6]
DS-SabioY oligo 1	AACTTATTGTAAACTTTTCATTTC TTAAAGTTTACAATGGTGCT	Top strand oligo containing *SabioY* wildtype sequence	[6]
DS-SabioY oligo 2	AGCACCATTGTAAACTTTAAGAAA TGAAAAGTTTACAATAAGTT	Bottom strand oligo containing *SabioY* wildtype sequence	[6]

Table S7. Bacterial strains utilised within this study.

Strain	Description	Function	Source
E. coli DH5α	*sup*EΔ*lac*169 (p80*lac*ZΔM15) *hsdR17* *rec*A1 *end* AA1 *gyr*A96 *thi*-1 *rel*A1	Plasmid manipulation	New England Biolabs, MA, USA
E. coli BL21-CodonPlus(DE3)-RIPL	*E. coli B F^- ompT DhsdS (rB^-mB^-) dcm^+Tet^R gal λ(DE3) endA Hte [argU proL Cm^R] [argU ileY leuW Strep/$Spec^R$]*	Protein expression strain	Aglient Technologies, CA, USA
S. aureus RN4220	Restriction negative derivative of 8325-4	Competent for transformation	University of Adelaide
S. aureus NCTC 8325	Methicillin sensitive *S. aureus* with reference genome	Parental *S. aureus* strain to *S. aureus* NCTC8325-B7	American Tissue Culture Collection
S. aureus NCTC8325-B7	*S. aureus* NCTC8325 after 18 days passage BPL199 (*birA* D200E)	Strain containing D200E mutation	This study

Table S8. Specific strains used for the *in vivo* beta-galactosidase reporter assay.

Strain	Integrations	Description	Source
JD26186 *birA::CAT*	*bioC::KanR birA::CAT*	JD28186 strain with N-terminal CAT cassette insertion (knockout)of its endogenous *birA*	[6]
JD26186 *birA::CAT-SabioO-SaBPL*	*bioC::KanR birA::CAT (SabioO-lacZ)HK(placUV5-SaBPL) λ*	JD26186 birA::CAT strain with SaBioO lacZ reporter chromosomally integrated at HK022 att site, and placUV5-SaBPL(wildtype) cassette chromosomally integrated at lambda att site.	[6]
JD26186 *birA::CAT-SabioY-SaBPL*	*bioC::KanR birA::CAT (SabioY-lacZ)HK (placUV5-SaBPL) λ*	JD26186 birA::CAT strain with SaBioY-lacZ reporter chromosomally integrated at HK022 att site and placUV5-SaBPL(wildtype) cassette chromosomally integrated at lambda att site.	[6]
JD26186 *birA::CAT-SabioO-SaBPL D200E*	*bioC::KanR birA::CAT (SabioO-lacZ)HK (placUV5-SaBPL D200E) λ*	JD26186 birA::CAT strain with SaBioO-lacZ reporter chromosomally integrated at HK022 att site, and plac-UV5-SaBPL (D200E) cassette chromosomally integrated at lambda att site.	This study
JD26186 *birA::CAT-SabioY-SaBPL D200E*	*bioC::KanR birA::CAT (SabioY-lacZ)HK (placUV5-SaBPL D200E) λ*	JD26186 birA::CAT strain with SaBioY-lacZ reporter chromosomally integrated at HK022 att site, and plac-UV5-SaBPL (D200E) cassette chromosomally integrated at lambda att	This study

Table S9. Plasmids utilised in this study.

Plasmid	Description	Source
pGEMT-*Sa*BPL(6xHis)	pGEMT plasmid containing *saBPL* with 6x his-tag	[7]
pGEMT-*Sa*BPL-D200E-(6xHis)	pGEMT plasmid containing *saBPL* D200E with 6x his-tag	This study
pIT4_TL_152002	Chromosomal integration plasmid (λ-attP, Tc^R, R6Kγ ori, *ccdB,* pUC ori)	[5]
pIT4_TL_SaBPL (D200E)	plac-UV5 fused with SaBPL (D200E) sequence cloned into pIT4_TL_152002	This study

References

1. Pendini, N. R.; Yap, M. Y.; Polyak, S. W.; Cowieson, N. P.; Abell, A.; Booker, G. W.; Wallace, J. C.; Wilce, J. A.; Wilce, M. C. J., Structural characterization of *Staphylococcus aureus* biotin protein ligase and interaction partners: An antibiotic target. *Protein Sci.* **2013,** *22* (6), 762-773.
2. Lee, K. J.; Tieu, W.; Blanco-Rodriguez, B.; Paparella, A. S.; Yu, J.; Hayes, A.; Feng, J.; Marshall, A. C.; Noll, B.; Milne, R.; Cini, D.; Wilce, M. C. J.; Booker, G. W.; Bruning, J. B.; Polyak, S. W.; Abell, A. D., Sulfonamide-Based Inhibitors of Biotin Protein Ligase as New Antibiotic Leads. *ACS Chem Biol* **2019,** *14* (9), 1990-1997.
3. Satiaputra, J.; Sternicki, L. M.; Hayes, A. J.; Pukala, T. L.; Booker, G. W.; Shearwin, K. E.; Polyak, S. W., Native mass spectrometry identifies an alternative DNA-binding pathway for BirA from Staphylococcus aureus. *Sci Rep* **2019,** *9* (1), 2767.
4. Benton, B. M.; Zhang, J. P.; Bond, S.; Pope, C.; Christian, T.; Lee, L.; Winterberg, K. M.; Schmid, M. B.; Buysse, J. M., Large-scale identification of genes required for full virulence of Staphylococcus aureus. *J Bacteriol* **2004,** *186* (24), 8478-89.
5. St-Pierre, F.; Cui, L.; Priest, D. G.; Endy, D.; Dodd, I. B.; Shearwin, K. E., One-step cloning and chromosomal integration of DNA. *ACS Synthet. Biol.* **2013,** *2* (9), 537-541.
6. Satiaputra, J.; Eijkelkamp, B. A.; McDevitt, C. A.; Shearwin, K. E.; Booker, G. W.; Polyak, S. W., Biotin-mediated growth and gene expression in Staphylococcus aureus is highly responsive to environmental biotin. *Appl Microbiol Biotechnol* **2018,** *102* (8), 3793-3803.
7. Pendini, N. R.; Polyak, S. W.; Booker, G. W.; Wallace, J. C.; Wilce, M. C. J., Purification, crystallization and preliminary crystallographic analysis of biotin protein ligase from *Staphylococcus aureus*. *Acta Crystallogr. Sect. F Struct. Biol. Cryst. Commun.* **2008,** *64*, 520-523.
